# Emotional eating is learned not inherited in children, regardless of obesity risk

**DOI:** 10.1111/ijpo.12428

**Published:** 2018-06-21

**Authors:** M. Herle, A. Fildes, C. H. Llewellyn

**Affiliations:** ^1^ Institute of Child Health University College London London UK; ^2^ School of Psychology University of Leeds Leeds UK; ^3^ Department of Behavioural Science and Health University College London London UK

**Keywords:** Childhood obesity, eating behaviour, emotional eating, twin research

## Abstract

**Background:**

Emotional over‐eating (EOE) and emotional under‐eating (EUE) are common behaviours that develop in early childhood and are hypothesised to play a role in weight status. Data from a British twin cohort demonstrated that environmental, rather than genetic, factors shape individual differences in both behaviours in early childhood.

**Objective:**

The aim of this current study was to replicate this finding in a subsample (*n* = 398) of 4‐year‐old twins selected for high or low risk of obesity from another population‐based cohort of British twins (the Twins Early Development Study).

**Methods:**

Parental ratings of child EOE and EUE were analysed using genetic model fitting.

**Results:**

Genetic influence was not significant, while shared environmental factors explained 71% (52–79%) of the variance in EOE and 77% (62–85%) in EUE. The two behaviours correlated positively (*r* = 0.53, 95% CI: 0.44, 0.61), and about two‐thirds of the shared environmental factors influencing EOE and EUE were the same (*r*_C_ = 0.67, 95% CI: 0.51, 0.85).

**Conclusions:**

Emotional eating in childhood is shaped by the home family environment; parents are therefore promising intervention targets.

## Introduction

Stress and negative emotions can have differential impacts on appetite, with some individuals experiencing decreased, and some increased, desire to eat 1. These behaviours, termed emotional over‐eating (EOE) and emotional under‐eating (EUE), have been implicated in the development of childhood weight 2 and eating disorders 3. Data from the Gemini twin study (2402 families; 4804 twins) showed that both EOE 4 and EUE 5 are shaped primarily by the shared family environment, not by genes, in early childhood. This contrasts strongly with other eating behaviours found to be under substantial genetic control in early life 6, 7. Replication is an essential part of scientific progress 8, and here we aimed to verify the strong environmental component to child EOE and EUE in a sample selected for high and low obesity risk.

## Methods

Data were from a subsample of the Twins Early Development Study 9, which included 214 pairs of twins from ‘obese’ and ‘lean’ families. One hundred sets of overweight or parents with obesity [maternal body mass index {BMI} > 28.5, paternal BMI > 25] and 114 healthy weight parents (both parents' BMI < 25) were identified and matched by social class, indexed using parental occupation. Families were visited at home for data collection when the twins were 4 years old 10. All anthropometrics were measured by the researchers. Parents indicated the ethnicity of their children by choosing between White and non‐White.

Parents reported on their children's emotional eating using the Child Eating Behaviour Questionnaire 11 subscales for EOE (four items, example item: ‘My child wants to eat more when irritable’) and EUE (four items, example item: ‘My child wants to eat less when sad’). BMI‐standard deviation (SD) scores were calculated for the children using British 1990 growth reference data 12, 13.

## Analyses

Prior to analyses, EOE and EUE scores were regressed by gestational age, age at measurement and sex. The twin method compares the twin pair similarity of mono‐zygotic (MZ) and di‐zygotic (DZ) twin pairs to decompose variation in EOE and EUE into three latent factors – additive genetic influences (A, heritability), shared (C) and non‐shared environmental influences (E). A great difference between MZ and DZ twin similarity implies significant contribution from genetic factors, whereas similar twin correlations, regardless of zygosity, indicate a contribution of the shared environment. Here, in addition, a bivariate correlated factors model was used to derive aetiological correlations between EOE and EUE, indicating the extent to which the genetic (r_A_), shared environmental (r_C_) and unique environmental (r_E_) influences underlying the two behaviours are the same 14. Analyses use maximum likelihood structural equation modelling and were carried out using OpenMx 15, a statistical package in R.

## Results

Data for EOE and EUE were available for 394 twins (197 pairs) when the children were 4 years old (Table 1). EOE and EUE correlated positively [*r* = 0.53, 95% confidence interval {CI}: 0.44, 0.61].

**Table 1:** Descriptive statistics for the TEDS analysis sample

Twin pairs	% or mean (SD)
Total	197 pairs (394 children)
Zygosity	
MZ pairs*	89 (45.2)
DZ pairs*	108 (54.8)
Sex	
Males	177 (44.9)
Females	217 (55.1)
Gestational age (weeks)	36.63 (2.61)
Age at measurement of EOE and EUE (years)	4.41 (0.35)
Emotional over‐eating (EOE)	1.84 (0.53)
Emotional under‐eating (EUE)	2.84 (0.82)
Child BMI‐SDS	0.45 (1.19)
Child weight status†	
Normal weight	319 (81)
Overweight	55 (14)
Obese	16 (4)
Missing	4 (1)
Maternal age at twin birth (years)	34.80 (4.36)
Ethnicity	
White	368 (93.4)
Non‐White	26 (6.6)
Socio‐economic status‡	
High	76 (38.6%)
Intermediate	78 (39.6%)
Low	27 (13.7%)
Missing	16 (8.1%)

* DZ, di‐zygotic; MZ, mono‐zygotic.

† Weight status was derived using the International Obesity Task Force reference standards 16.

‡ Families were grouped by the occupation of the highest earner per family: high (higher and lower managerial and professional occupations), intermediate (intermediate occupations, small employers and own account workers – self‐employed with no employees) and lower occupational classifications [lower supervisory and technical occupations, {semi‐} routine occupations, never worked and long‐term unemployed].

BMI‐SDS, body mass index‐standard deviation scores; SD, standard deviation; TEDS, Twins Early Development Study.

The full ACE model fitted the data well compared with the saturated model which estimates means and variances freely without any constraints (Δχ^2^ = 6.134, Δ Df = 17, *p* = 0.99). Heritability was not significant for either EOE (A = 0.03, 95% CI: 0.00, 0.25) or EUE (A = 0.04, 95% CI: 0.00, 0.21), but CIs were wide. Shared environmental influences were strong for both EOE (C = 0.71, 95% CI: 0.52, 0.79) and EUE (C = 0.77, 95% CI: 0.62, 0.85). The contributions from non‐shared environmental factors were moderate (EOE: E = 0.26, 95% CI: 0.19, 0.34; EUE: E = 0.19, 95% CI: 0.14, 0.25).

**Figure 1:**
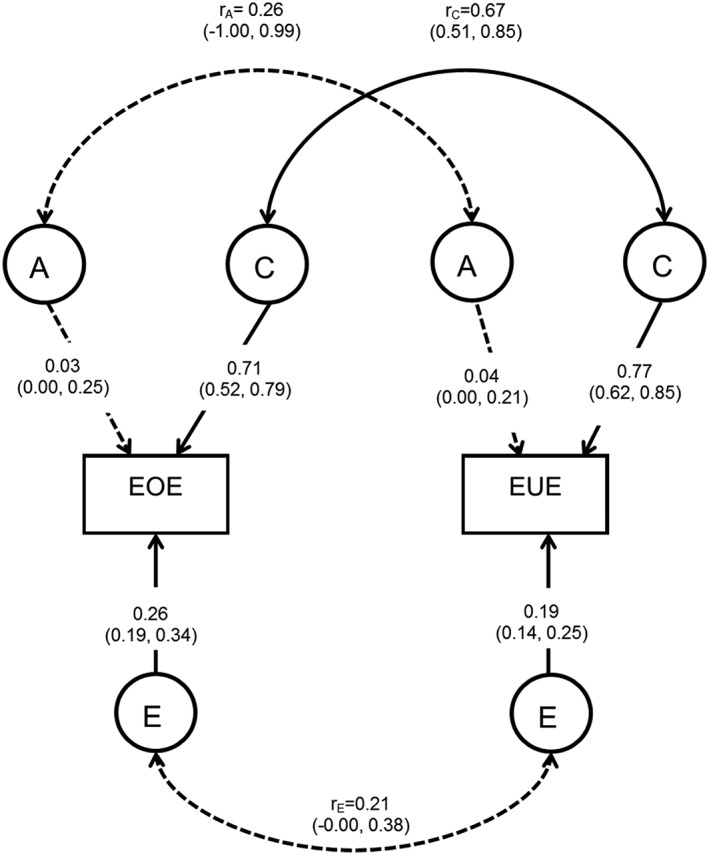
Correlated factors model of emotional over‐eating and emotional under‐eating. The rectangular boxes represent the measured phenotype [emotional over‐eating {EOE} and emotional under‐eating {EUE}] using the Child Eating Behaviour Questionnaire at 4 years of age. The circles indicate the latent factors: genetic effects (A), shared environmental effects (C) and non‐shared environmental effects (E). The straight single‐headed arrows reflect pathways with the variance explained by each latent factor [including 95% confidence intervals {CIs}]. The non‐significant A paths, with 95% CIs including 0, are represented as dotted lines. The aetiological correlations are shown on the curved double‐headed arrows. These indicate the extent of common genetic (r_A_), shared environmental (r_C_) and non‐shared environmental (r_E_) influences across the two phenotypes. The non‐significant aetiological correlations (r_A_ and r_E_), with 95% CI crossing 0, are represented as dotted lines.

The aetiological correlations indicated that only shared environmental factors (r_C_ = 0.67, 95% CI: 0.51, 0.85) were common to both behaviours because neither genetic nor non‐shared environmental correlations were significant (r_E_ = 0.21, 95% CI: −0.00, 0.38; r_A_ = 0.26, 95% CI: −1.00, 0.99). Figure 1 shows a path diagram illustrating the results.

## Discussion

This twin study of the aetiology of EOE and EUE in early childhood replicated previous findings from the Gemini twin cohort. As previously found 5, EOE and EUE correlated positively (*r* = 0.53), indicating that some children tend to both over‐eat and under‐eat in response to stress. Previous research has suggested that an individual's appetitive response to stress might be due to the nature and intensity of the stress – consistent low level stress relating to over‐eating, whereas intense stress might lead to under‐eating 17. Results supported that both behaviours are learned in childhood, with the shared environment exerting the strongest effect. Genetic contributions were minimal and non‐significant. Furthermore, EOE and EUE had some shared aetiology, in line with previous findings; approximately two thirds of the shared environmental influences on each behaviour were the same (r_C_ = 0.67). Notably, this sample was selected to include families with varying degrees of obesity risk, increasing the heterogeneity and representativeness of the findings. The mean and SD of the BMI‐SD scores (mean = 0.45, SD = 1.19) indicated higher mean BMI than the UK population‐based reference data for children of this age and sex, as well as large weight variation in this sample. Limitations were the reduced sample size, leading to wide CIs, and the inclusion of only same‐sex DZ twin pairs, precluding testing for aetiological differences between boys and girls. Furthermore, parental weight is only one of many risk factors for child obesity and does not capture the full ‘picture’, and more research is needed to establish if the aetiology of EOE and EUE changes with developmental stage. The effect of stress on eating behaviour is complex, and a previous study has indicated a Gene–Environment interaction demonstrating that parents are more inclined to use maladaptive feeding strategies if their children are of increased genetic risk associated with emotional dysregulation 18.

In summary, this study supported that emotional eating is learned in early childhood, not inherited. Shared environmental factors, potentially parental feeding strategies such as emotional feeding 19, are key drivers of EOE and EUE regardless of a child's familial risk of obesity. These findings highlight family level factors as promising intervention targets for the prevention of emotional eating in children.

## Conflict of interest statement

No conflict of interest was declared.

## Author contributions

The authors' responsibilities were as follows: all authors (M. H., A. F. and C. L.) designed the research; M. H. performed the statistical analyses; and all authors wrote, read and approved the final manuscript.
